# Antiretroviral therapy related adverse effects: Can sub-Saharan Africa cope with the new “test and treat” policy of the World Health Organization?

**DOI:** 10.1186/s40249-017-0240-3

**Published:** 2017-02-15

**Authors:** Jobert Richie N. Nansseu, Jean Joel R. Bigna

**Affiliations:** 1 Department of Public Health, Faculty of Medicine and Biomedical Sciences of the University of Yaoundé I, PO Box 1364, Yaoundé, Cameroon; 2 Sickle Cell Disease Unit, Mother and Child Centre of the Chantal Biya Foundation, Yaoundé, Cameroon; 3 Department of Epidemiology and Public Health, Centre Pasteur of Cameroon, Yaoundé, Cameroon; 4 Faculty of Medicine, University of Paris Sud XI, Le Kremlin Bicêtre, Paris, France

**Keywords:** HIV/AIDS, Test and treat, 90-90-90, Antiretroviral therapy, Sub-Saharan Africa

## Abstract

**Background:**

Recent studies have shown that early antiretroviral therapy (ART) initiation results in significant HIV transmission reduction. This is the rationale behind the “test and treat” policy of the World Health Organization (WHO). Implementation of this policy will lead to an increased incidence of ART-related adverse effects, especially in sub-Saharan Africa (SSA). Is the region yet ready to cope with such a challenging issue?

**Main body:**

The introduction and widespread use of ART have drastically changed the natural history of HIV/AIDS, but exposure to ART leads to serious medication-related adverse effects mainly explained by mitochondrial toxicities, and the situation will get worse in the near future. Indeed, ART is associated with an increased risk of developing cardiovascular disease, lipodystrophy, prediabetes and overt diabetes, insulin resistance and hyperlactatemia/lactic acidosis. The prevalence of these disorders is already high in SSA, and the situation will be exacerbated by the implementation of the new WHO recommendations. Most SSA countries are characterized by (extreme) poverty, very weak health systems, inadequate and low quality of health services, inaccessibility to existing health facilities, lack of (qualified) health personnel, lack of adequate equipment, inaccessibility and unaffordability of medicines, and heavy workload in a context of a double burden of disease. Additionally, there is dearth of data on the incidence and predictive factors of ART-related adverse effects in SSA, to anticipate on strategies that should be put in place to prevent the occurrence of these conditions or properly estimate the upcoming burden and prepare an adequate response plan. These are required if we are to anticipate and effectively prevent this upcoming burden.

**Conclusion:**

While SSA would be the first region to experience the huge benefits of implementing the “test and treat” policy of the WHO, the region is not yet prepared to manage the consequential increased burden of ART-related toxic and metabolic complications. Urgent measures should be taken to fill the lacunae if SSA is not to become over-burdened by the consequences of the “test and treat” policy.

**Electronic supplementary material:**

The online version of this article (doi:10.1186/s40249-017-0240-3) contains supplementary material, which is available to authorized users.

## Multilingual abstracts

Please see Additional file 1 for translations of the abstract into the six official working languages of the United Nations.

## Background

According to the Joint United Nations Programme on HIV/AIDS (UNAIDS) global estimates, 38.7 million people were living with HIV in 2015, among whom 2.1 million became newly infected (including 150 000 children) and 1.1 million died from AIDS-related illnesses [1]. The HIV pandemic is to-date one of the most serious health threats the world has ever witnessed. Since the beginning of the epidemic, around 78 million people have become infected with HIV, and 35 million people have died from related complications [1]. For the specific case of sub-Saharan Africa (SSA), 6.5 to 19 million people were living with HIV in the region, with 410 000 to 960 000 new infections and 330 000 to 470 000 related deaths in 2015 [1].

Fortunately, the introduction and widespread use of highly active antiretroviral therapy (ART) have drastically changed the natural history of HIV/AIDS. Indeed, the longevity, prognosis and quality of life of people living with HIV/AIDS (PLWHA) have significantly improved over time. For instance, AIDS-related deaths have decreased by 45% between 2005 and 2015 [1]. Initiation of ART was initially based on the World Health Organization (WHO) clinical stages or CD4 cell count [2, 3]. But, these guidelines have recently been revised, following conclusive reports from randomized controlled trials which have shown a substantial benefit of early ART initiation with significant reduction in HIV transmission [4–6]. Based on these studies, the UNAIDS has set out an ambitious target for 2020. By this year, they would like 90% of people living with HIV to know their status, 90% of all people with diagnosed HIV infection to receive sustained ART, and 90% of all people receiving ART to have viral suppression [7]. In order to achieve this target, the WHO recommendations changed to a “test and treat” policy advocating that ART should be immediately started in all individuals diagnosed with HIV regardless of age or CD4 cell count [8].

By the end of 2015, almost 17 million people were already accessing ART globally and as a result of the policy shift to “test and treat”, an exponential increase in the number of people receiving ART can thus be expected in the near future [1]. However, continuous ART may lead to serious adverse effects mainly explained by mitochondrial toxicities. These include: lactic acidosis, pancreatitis, lipodystrophy, dyslipidaemias, insulin resistance and dysglycemias [9–16]. Accordingly, it can be anticipated that the burden of ART-related complications (both toxic and metabolic) will increase in the near future, especially in SSA which is the epicenter of the HIV pandemic globally. This gives rise to the important question of whether the region is sufficiently equipped and prepared to manage this impending burden. The present paper discusses the readiness of SSA countries in implementing the “test and treat/90-90-90” policies with regard to the upcoming increased burden of ART-mediated toxic and metabolic complications.

### Snapshot of challenges to implement the “test and treat/90-90-90” policies in Africa

Each “90” target is subjected to difficulties in its implementation in SSA. For the first “90”: 90% of all people living with HIV should know their HIV status, one can include without limitation, lack of HIV testing proposals by healthcare providers and low acceptance for HIV testing by people [17]. Actually, it is recognized that only 20% of PLWHA in SSA know their HIV seropositive status [18]. One of the greatest challenges in Africa would be to implement easily accessible HIV counselling and voluntary HIV testing. This is due to limited financial resources, medical supplies, and weak health systems.

For the second “90”: 90% of people diagnosed with HIV infection should receive sustained ART, difficulties can include low rate of linkage to care, low rate of early ART initiation after HIV seropositive screening, and inadequate/insufficient stocks of ART to provide continuous medication to PLWHA [17, 19]. A study in SSA showed for instance that only 12% of diagnosed-PLWHA were linked to HIV/AIDS care after home-based HIV counselling and testing [20].

For the third “90”: 90% of all people receiving ART should have viral suppression, absence of proven methods to ensure long time adherence to ART and retention in care for all HIV-infected people, inadequate stocks of ART, and weak early warning indicators for HIV drug resistance can negatively impact this objective in Africa [17, 21–25]. One can also include as transversal impediments, lack of required financial resources for HIV programs and limited supportive health system infrastructures in resource-constrained settings.

### Snapshot of the current burden of medication-related adverse effects of continuous ART in SSA

There is growing evidence of a significant rise in cardiovascular disease (CVD) occurrence in PLWHA, more so to those ART-exposed [26]. Studies in SSA signal to an increased incidence of hypertension, diabetes mellitus (DM), renal disease and stroke among ART-naïve PLWHA as well as those on continuous ART [27–34]. Moreover, lipid disorders are very common in this population: the overall prevalence of metabolic syndrome turns around 8.7–58% in Africa [14, 35, 36]. Other studies have specifically identified other known CVD risk factors: higher low-density lipoproteins levels, lower high-density lipoproteins levels, and higher apolipoprotein B/apolipoprotein A ratio [11, 27].

In addition, ART has been implicated as a risk factor for developing prediabetes and overt diabetes mellitus [37]. In some parts of SSA, the prevalence of dysglycemias is almost 40% in ART-exposed PLWHA [12, 15, 38]. More studies are needed to correctly estimate this risk of CVD and metabolic syndrome, and identify factors that can predict their development.

The prevalence of lipodystrophy in Africa reaches 69.9% and the incidence, 41.6% among PLWHA exposed to ART [16], especially nucleoside analogue reverse transcriptase inhibitors (NRTI)-containing regimen [10]. Lipodystrophy is known to be associated with older age and longer duration on therapy, and has been linked with a decreased quality of life, alterations in lipid, glucose and insulin metabolisms, and an increased risk of DM and CVD [16, 39].

Due to their ability to inhibit polymerase-γ, NRTIs are associated with several mitochondrial toxicities, including life-threatening lactic acidosis. The prevalence of hyperlactatemia is very high in SSA, varying between 17.1 and 83.2% among PLWHA on ART. Once symptomatic, hyperlactatemia is associated with weakness, skin rashes, tachypnea, and can lead to death [9, 13, 40–42]. However, we recognize that this prevalence could be lower, considering that the new first line ART recommended by the WHO does include ART with a lower toxicity profile than previous regimen (removing stavudine for example).

### Readiness of SSA countries to implement the new policies of the WHO and UNAIDS: myth or reality?

Countries of the SSA region are resource-poor settings with very weak health systems and poor quality of health services [43–45], especially in the respect of implementing the ‘test and treat’/90-90-90 policies [17, 19]. Health facilities are not found everywhere, particularly in remote and underserved rural areas. When these health centres do exist, they are not always accessible due to non-existent or unpassable roads. There is a crucial lack of (qualified) health care personnel, with some areas not having seen even a nurse. Under these circumstances, who will test the population for HIV and follow-up those found infected? Where are they going to be followed-up, given the insufficient number of health facilities and personnel? With 60% of the African population living in rural areas and considering the extreme shortage of health facilities and professionals in rural Africa, there is urgent need to address these gaps with the regard of the “test and treat” implementation in SSA. In addition to the current shortfall of personnel and facilities necessary to the full implementation of the 90-90-90 policy, and with the expected increase in metabolic disorders secondary to longer ART durations, there is a great need for equipment, and more importantly, adequate numbers of suitably qualified physicians and nurses to closely monitor PLWHA. But these are not currently available. For instance, CVD and DM remain poorly investigated and managed in SSA [46–49]. Moreover, SSA countries are still beset with infectious diseases which continue to exact a heavy tool of illness and death in the region [50]. What’s worse, non-communicable diseases are becoming common in these countries, leading thus to the double burden of disease [50]. With the projected increase in adverse events following initiation of ART in all PLWHA, the current personnel, facilities, equipment, and funding shortfalls will be greatly exacerbated in SSA if some key measures are not implemented.

To date, there are no clear estimates on the incidence of and risk factors for metabolic complications of ART among HIV-infected people living in SSA, children specifically [37, 39]. Therefore, it is difficult to predict what proportion of PLWHA in SSA will develop ART-related adverse effects and develop adequate action plans to counter them. Since the risk factors for developing these adverse effects are not known, measures to prevent them cannot be formulated. Additionally, there is no clear evidence on measures that might prevent PLWHA on long-term continuous ART from developing ART-related adverse effects, this by acting on identified risk factors. Studies are therefore urgently required to fill these gaps and help in designing effective strategies to curtail the expected increase in ART-related adverse effects in SSA following adoption of the new HIV/AIDS recommendations.

Notwithstanding these fears, evidence suggests that WHO/UNAIDS goals may be achievable in some SSA nations. The SEARCH study was conducted in 32 communities in Uganda and Kenya and a total of 334 540 people were enrolled. Participants were randomized to either receive standard HIV services or an expanded testing and treatment program. Preliminary results show that 90% of adults accepted the offer of testing, 93% of those diagnosed with HIV stayed in medical care for at least 6 months, and 92% of those in care had a fully suppressed viral load [51]. Likewise, preliminary results from the PopART study which involved 21 communities in South Africa and Zambia with a cumulative population of 1.2 million people revealed that an estimated 87% of women and 79% of men with HIV knew their status, but just 65 and 62% of diagnosed men and women respectively went on therapy. These numbers are steadily increasing as the study continues [51, 52]. However, the prevalence and incidence of ART-related adverse effects were not looked into in these studies. Also, the researchers have been silent on strategies to identify and handle these complications. The development of evidence-based policy is urgently needed in this regard.

## Conclusion

Although it is unequivocally clear that early ART initiation in the course of HIV infection will be beneficial at both the individual and population levels, SSA appears not yet ready to cope with the requirements and consequences of implementing the “test and treat” strategy in an efficient manner. Lack of organized health systems, health facilities and qualified workforce may jeopardize attainment of the WHO/UNAIDS goals. Furthermore, adequate equipment and medicines will need to be made accessible and affordable for all those PLWHA who will develop ART-related adverse effects. Technical and financial aids will be required for SSA countries to develop systems and capacities to fill their gaps and strengthen their weaknesses. Studies are urgently warranted to study the incidence of and risk factors for ART-related complications. These will enable countries in SSA to draw strategies to tackle them effectively.

## Additional file

Additional file 1: Multilingual abstracts in the six official working languages of the United Nations. (PDF 680 kb)

## Abbreviations

AIDS: Acquired immunodeficiency syndrome
ART: Antiretroviral therapy
CVD: Cardiovascular disease
DM: Diabetes mellitus
HIV: Human immunodeficiency virus
NRTI: Nucleoside analogue reverse transcriptase inhibitors
PLWHA: Person living with HIV/AIDS
SSA: Sub-Saharan Africa
UNAIDS: Joint United Nations Programme on HIV/AIDS
WHO: World Health Organization
