# Supplementary material for: Antiretroviral therapy related adverse effects: Can sub-Saharan Africa cope with the new “test and treat” policy of the World Health Organization?
Source: Infect Dis Poverty. 2017 Feb 15;6:24. doi: 10.1186/s40249-017-0240-3 (PMC5310075; doi:10.1186/s40249-017-0240-3)
Supplement: Additional file 1: — Multilingual abstracts in the six official working languages of the United Nations. (PDF 680 kb) [file 40249_2017_240_MOESM1_ESM.pdf]

Translation of the abstract into the six official working languages of the United Nations

الآثار السلبية المرتبطة بالعلاج بمضادات الفيروسات القهقرية: هل بإمكان افريقيا جنوب الصحراء الكبرى مواكبة خطة منظمة الصحة العالمية "اختبر وعالج"

جوبرت ريشي نانسو، جين جويل بيغنا

ملخص الدراسة

خلفية الموضوع: أظهرت الدراسات الحديثة أن البدء المبكر بالعلاج بمضادات الفيروسات القهقرية يؤدي إلى تخفيض إنتقال عدوى مرض نقص المناعة المكتسبة بشكل كبير. وهذا هو الأساس المنطقي وراء سياسة منظمة الصحة العالمية "للإختبار والعلاج". تنفيذ هذه الخطة سيقود إلى ازدياد فرص حدوث التأثيرات الجانبية للعلاج بمضادات الفيروسات القهقرية وخصوصاً في افريقيا جنوب الصحراء الكبرى. فهل المنطقة لاتزال على استعداد للتعامل مع مثل هذه القضية الصعبة؟

الهيكل الرئيسي: لقد غير الإستخدام الواسع للعلاج بمضادات الفيروسات القهقرية التاريخ الطبيعي لمرض نقص المناعة المكتسبة (الإيدز) بشكل جذري. إلا أن الخضوع للعلاج مضادات الفيروسات القهقرية قد أدى إلى أثارسلبية خطيرة متعلقة بهذا الدواء والتي يمكن شرحها بالإشارة إلى سميات الميتوكوندريا (العضيات) وسيزداد الوضع سوءا في المستقبل القريب. فقد ارتبط استخدام العلاج بمضادات الفيروسات القهقرية بزدياد خطر الإصابة بأمراض القلب والأوعية والحثل الشحمي وماقبل مرض السكري ومرض السكري ومقاومة الانسولين واضطرابات حمض اللاكتيك. هذه الاضرابات منتشرة مسبقا في افريقيا جنوب الصحراء الكبرى وسيتفاقم الوضع من خلال تنفيذ توصيات منظمة الصحة العالمية الجديدة. تتميز معظم بلدان افريقيا جنوب الصحراء الكبرى بنسبة الفقر (المدقع) وأنظمة الصحة الضعيفة وتدني الخدمات الصحية وعد إمكانية الوصول إلى المرافق الصحية ونقص بعدد العاملين في مجال الصحة (المؤهلين) ونقص بالمعدات الملائمة وعدم المقدرة على الحصول على الدواء وعلى تكلفته وعبئ العمل الكبير في سياق العبئ المزدوج للمرض. بالإضافة الى ذلك هناك ندرة في البيانات عن الحالات و عن عوامل التنبؤ للآثار السلبية المرتبطة بالعلاج بمضادات الفيروسات القهقرية في جنوب الصحراء الكبرى والتي قد تساعد بوضع الاستراتيجيات لمنع حدوث هذه الحالات أو ربما لتقيم العبئ القادم وإعداد خطة استجابة ملائمة. هناك حاجة لهذه البيانات إذا أردنا ان نقيم و نمنع هذا العبئ القادم على نحو فعال.

الخاتمة: على الرغم من أن جنوب الصحراء الكبرى ستكون المنطقة الأولى لتجربة الفوائد الكبيرة لتنفيذ خطة منظمة الصحة العالمية "اختبر وعالج" الا أن المنطقة ليست مستعدة بعد للتعامل مع زيادة العبئ المترتب على المضاعفات السامة والأيضية المرتبطة باستخدام العلاج بمضادات الفيروسات القهقرية. وبالتالي هناك حاجة ماسة لاتخاذ تدابير عاجلة لملئ الثغرات كي لاتصبح منطقة افريقيا جنوب الصحراء مثقلة أكثر بسبب عواقب استخدام خطة منظمة الصحة العالمية.

Translated from English version into Arabic by Randa82, through

TRANSLATORS
WITHOUT BORDERS

抗逆转录病毒疗法的相关不良反应：撒哈拉以南非洲地区能否应对世界卫生组织新的“检测与治疗”的政策？

Jobert Richie N. Nansseu, Jean Joel R. Bigna

摘要

引言：最近研究表明，早期抗逆转录病毒疗法（ART）可显著降低艾滋病传播。这是世界卫生组织“检测和治疗”政策背后的理论基础。这一政策的实施将导致抗逆转录病毒治疗的相关不良反应发生率增加，尤其是在撒哈拉以南非洲（SSA）。该地区是否已准备好应对这样一个具有挑战性的问题？

正文：ART 的引进和广泛使用已彻底改变了 HIV/AIDS 自然发展过程，但 ART 会引起主要由线粒体毒性引起的严重的 ART 药物相关不良反应，并且在不久的将来情况会变得更糟。事实上，ART 与患心血管疾病、脂肪代谢障碍、糖尿病前期和显性糖尿病、胰岛素抵抗和高乳酸血症或乳酸性酸中毒等的风险增加相关。在撒哈拉以南非洲上述疾病患病率已很高，WHO 新的实施建议将加剧这一情况。大多数撒哈拉以南非洲国家的特点是（极端）贫困、卫生系统非常薄弱、卫生服务不健全和质量低、卫生设施无法获取、缺乏（合格的）卫生人员，同时设备缺乏、交通不便、买不起药而且在患有多重疾病下从事繁重体力劳动。另外，缺乏关于 ART 相关不良反应发病率和预测因素的数据，因此无法提出预防这些情况发生或评估即将到来疾病负担的应对策略和适当的响应计划。如果我们要预测并有效繁殖即将到来的负担，以上都是必须具备的。

结论：尽管撒哈拉以南的非洲国家将是享受 WHO“检测与治疗”政策巨大利益的第一个区域，但该区域尚未做好如何应对 ART 相关毒性和代谢并发症带来巨大负担的准备，应采取紧急措施来填补这些漏洞，以防止该区域国家不堪重负于“检测与治疗”政策带来的副作用。

Translated from English version into Chinese by Yin-Long Li, edited by Pin Yang

CHINESE CENTER FOR DISEASE CONTROL AND PREVENTION
NATIONAL INSTITUTE OF PARASITIC DISEASES

Effets indésirables associés au traitement antirétroviral: l'Afrique subsaharienne peut-elle donner suite à la nouvelle politique «de dépistage et de traitement» de l'Organisation mondiale de la Santé?

Jobert Richie N. Nansseu, Jean Joel R. Bigna

Résumé

Contexte: Des études récentes ont montré que l'amorce rapide d'un traitement antirétroviral (TAR) entraîne une diminution importante de la transmission du VIH. C'est la raison d'être de la politique «de dépistage et de traitement» de l'Organisation mondiale de la Santé (OMS). La mise en œuvre de cette politique se traduira par une incidence accrue des effets indésirables imputables au TAR, notamment dans l'Afrique subsaharienne. Toutefois, la région est-elle prête à faire face à ce grand enjeu?
Partie principale: L'introduction du TAR et sa large utilisation ont énormément changé l'évolution naturelle du VIH/sida. Cependant, l'exposition au TAR se traduit par de graves effets indésirables associés au traitement principalement en raison de toxicités mitochondriales, et la situation empirera dans un avenir proche. En effet, le TAR est associé à un risque accru d'apparition de la maladie cardiovasculaire, de la lipodystrophie, du prédiabète et du diabète clinique, de l'insulinorésistance et de l'hyperlactatémie/de l'acidose lactique. La prévalence de ces maladies est déjà élevée dans l'Afrique subsaharienne, et la situation sera exacerbée par la mise en action des nouvelles recommandations de l'OMS. La plupart des pays de l'Afrique subsaharienne sont caractérisés par une pauvreté (extrême), des systèmes de santé très défaillants, des services de santé insuffisants et de qualité médiocre, l'inaccessibilité aux établissements de santé existants, le manque de personnel de santé (qualifié), le manque de matériel adéquat, l'inaccessibilité aux médicaments et l'inabordabilité de ceux-ci, et la charge de travail élevée dans un contexte de double fardeau de la maladie. De plus, il manque des données sur l'incidence et les prédicteurs des effets indésirables associés au TAR dans l'Afrique subsaharienne pour prévoir des stratégies qui devraient être mises en place afin d'empêcher la survenue de ces situations ou d'estimer correctement le fardeau à venir et de préparer un plan d'intervention adapté. Ce sont des éléments indispensables si nous avons à anticiper et à prévenir efficacement ce fardeau imminent.
Conclusion: Même si l'Afrique subsaharienne est la première région à connaître les bienfaits énormes de l'application de la politique «de dépistage et de traitement» de l'OMS, la région n'est pas encore prête à gérer le fardeau indirect accru des complications toxiques et métaboliques liées au TAR. Il faudra prendre des mesures urgentes pour combler les lacunes si on veut éviter que l'Afrique subsaharienne ne se retrouve accablée par les conséquences de la politique «de dépistage et de traitement».

Translated from English version into French by Vicky Victoire, through

TRANSLATORS
WITHOUT BORDERS

Негативные последствия антиретровирусной терапии: может ли Чёрная Африка справиться с новым подходом «тестировать и лечить» Всемирной организации здравоохранения?

Жобер Ричи Н. Нанссеу (Jobert Richie N. Nansseu), Джин Джоел Р. Бигна (Jean Joel R. Bigna)

Аннотация

Краткое описание. Недавние исследования показали, что раннее начало антиретровирусной терапии (АРТ) приводит к значительному снижению передачи ВИЧ-инфекции. На этом основан подход «тестировать и лечить» Всемирной организации здравоохранения (ВОЗ). Внедрение этого подхода приведёт к росту связанных с АРТ нежелательных отрицательных воздействий, особенно в Чёрной Африке. Готов ли этот регион справиться с такой трудной проблемой?
Содержание. Внедрение и широкое применение АРТ радикально изменило естественную динамику ВИЧ/СПИДа. Однако, АРТ ведёт к серьёзным негативным побочным эффектам, вызванным лекарственными средствами, главным образом к митохондриальной токсичности; и ситуация в ближайшем будущем будет ухудшаться. Более того, АРТ связан с увеличенным риском развития сердечно-сосудистых заболеваний, липодистрофии, предрасположенности к диабету и сахарному клиническому диабету, невосприимчивости к инсулину, гиперлактатемии и лактоацидозу. В Чёрной Африке уже отмечено высокое распространение этих болезней, и внедрение новых рекомендаций ВОЗ обострит положение. Для большинства стран Чёрной Африки характерны (чрезвычайная) бедность, слаборазвитые системы здравоохранения, неадекватное и низкое качество медицинского обслуживания, недоступность существующих лечебных учреждений, нехватка (квалифицированных) медицинских

кадров, недостаток нужного оборудования, сложность получения и дороговизна лекарств, сильная загруженность в условиях двойной нагрузки от заболеваний. Помимо этого, в Чёрной Африке недостаточно данных о частоте заболеваемости и прогностических факторах для связанных с АРТ нежелательными воздействиями для того, чтобы заранее разработать стратегии, которые следует применять для предотвращения подобных болезней или надлежащим образом оценить предстоящую нагрузку и подготовить соответствующий план действий. Все это необходимо делать, если мы прогнозируем и собираемся эффективно бороться с этими ожидающими нас в будущем трудностями.
Заключение. В то время как Чёрная Африка является первым регионом, которому подход ВОЗ «тестировать и лечить» принесёт огромную пользу, этот регион ещё не готов к тому, чтобы справиться с последующими возросшими трудностями, которые будут вызваны связанными с АРТ токсичными и метаболическими осложнениями. Следует принять срочные меры по устранению упущений, чтобы Чёрная Африка не пострадала от последствий подхода «тестировать и лечить».

Translated from English version into Russian by Natalia Potashnik, through

TRANSLATORS WITHOUT BORDERS

Efectos adversos relacionados con la terapia antiretroviral: Puede el África sub Sahariana hacer frente a la nueva política de "prueba y tratamiento" de la Organización Mundial de la Salud?

Jobert Richie N. Nansseu, Jean Joel R. Bigna

Resumen
Antecedentes: Recientes estudios han demostrado que el inicio temprano de una terapia antiretroviral (TARV) tiene como resultado una significativa reducción en la transmisión del VIH. Este es el motivo principal detrás de la política de "prueba y tratamiento de la "Organización Mundial de la Salud (OMS). La implementación de esta política creará a una mayor incidencia de los efectos adversos relacionados con TARV, - en África Sub Sahariana (SSA). Está ya lista la región para hacer frente a tal desafío?
Cuerpo Principal: La introducción y el amplio uso de la TARV ha cambiado de manera drástica la historia natural del VIH/SIDA, pero la exposición a la TARV lleva a serios efectos adversos relacionados con la medicación que se explican principalmente por la toxicidad mitocondrial y la situación empeorará en el futuro próximo. De hecho, la TARV está asociada con un mayor riesgo de desarrollar enfermedades cardiovasculares, lipodistrofia, pre diabetes y diabetes manifiesta, resistencia a la insulina e hiperlactatemia/ acidosis láctica. La prevalencia de estos trastornos ya es alta en SSA, y la situación empeorará debido a la implementación de las nuevas recomendaciones de la OMS. La mayoría de los países del SSA se caracterizan por extrema pobreza, sistemas de salud muy débiles, servicios de salud de baja calidad e inadecuados, inaccesibilidad a las instalaciones de salud existentes, falta de personal de salud calificado, falta de equipos adecuados, medicinas inaccesibles e inasequibles y carga pesada en un contexto de la doble carga de la enfermedad. Adicionalmente, en la SSA hay escases de datos sobre la incidencia y factores predictivos de los afectos adversos que se relacionan con la TARV, para anticipar estrategias que deben implementarse para prevenir que se presenten estas condiciones o estimar adecuadamente la futura carga y preparar un plan de respuesta que sea adecuado. Esto se requiere si queremos anticipar y prevenir de forma efectiva esta futura carga.
Conclusión: Pese a que SSA sería la primera región que experimente los inmensos beneficios de implementar la política de "prueba y tratamiento" de la OMS, la región no está aún preparada para manejar el peso mayor como consecuencia de las complicaciones metabólicas y tóxicas relacionadas con la TARV. Se deben tomar medidas urgentes para llenar los vacíos si no se quiere que la SSA se vea sobrecargada por las consecuencias de la política de "prueba y tratamiento".

Translated from English version into Spanish by Doloritas, through

TRANSLATORS WITHOUT BORDERS
